# Clear cell carcinoma of the ovary: Is there a role of histology-specific treatment?

**DOI:** 10.1186/1756-9966-31-53

**Published:** 2012-06-01

**Authors:** Masashi Takano, Hiroshi Tsuda, Toru Sugiyama

**Affiliations:** 1 Department of Obstetrics and Gynecology, National Defense Medical College, Tokorozawa, Saitama, 359-8513, Japan; 2 Department of Obstetrics and Gynecology, School of Medicine, Keio University, Shinano-machi 35, Shinjuku-ku, Tokyo, 160-8582, Japan; 3 Department of Obstetrics and Gynecology, Iwate Medical University, Morioka, Iwate, 020-8505, Japan

**Keywords:** Review, Ovarian cancer, Clear cell carcinoma, Surgical staging, Fertility-sparing, Chemotherapy, Molecular targeting agents

## Abstract

Several clinical trials to establish standard treatment modality for ovarian cancers included a high abundance of patients with serous histologic tumors, which were quite sensitive to platinum-based chemotherapy. On the other hand, ovarian tumor with rare histologic subtypes such as clear cell or mucinous tumors have been recognized to show chemo-resistant phenotype, leading to poorer prognosis. Especially, clear cell carcinoma of the ovary (CCC) is a distinctive tumor, deriving from endometriosis or clear cell adenofibroma, and response rate to platinum-based therapy is extremely low. It was implied that complete surgical staging enabled us to distinguish a high risk group of recurrence in CCC patients whose disease was confined to the ovary (pT1M0); however, complete surgical staging procedures could not lead to improved survival. Moreover, the status of peritoneal cytology was recognized as an independent prognostic factor in early-staged CCC patients, even after complete surgical staging. In advanced cases with CCC, the patients with no residual tumor had significantly better survival than those with the tumor less than 1 cm or those with tumor diameter more than 1 cm. Therefore, the importance of achieving no macroscopic residual disease at primary surgery is so important compared with other histologic subtypes. On the other hand, many studies have shown that conventional platinum-based chemotherapy regimens yielded a poorer prognosis in patients with CCC than in patients with serous subtypes. The response rate by paclitaxel plus carboplatin (TC) was slightly higher, ranging from 22% to 56%, which was not satisfactory enough. Another regimen for CCC tumors is now being explored: irinotecan plus cisplatin, and molecular targeting agents. In this review article, we discuss the surgical issues for early-staged and advanced CCC including possibility of fertility-sparing surgery, and the chemotherapy for CCC disease.

## Background

Clear cell adenocarcinoma (CCC) is a distinct entity from other epithelial ovarian carcinomas (EOC). CCC is thought to arise from endometriosis or clear cell adenofibroma, however, the origin of serous cyst adenocarcinoma (SCA) is thought to be Mullerian epithelium derived from either ovarian surface epithelium or fallopian tube (endosalpingiosis). CCC has specific biological and clinical behavior, compared with other histological types. However, in the studies used as evidence for recommended treatment as standard treatment of EOC, most of the enrolled patients were not clear cell histology, and these study results do not provide a scientific rationale for CCC. In this review, we summarize the treatment of CCC.

## Surgical treatment

The standard surgical treatment of patients with EOC is based on hysterectomy, bilateral salpingo-oophorectomy and partial omentectomy with peritoneal sampling and lymphadenectomy, and cytoreductive surgery is added especially for advanced cases. The surgical treatment of CCC is usually determined based on the guideline of EOC. In this section, we summarize the surgical treatment of CCC patients.

### Surgical staging

It has been reported that the incidence of lymph node metastasis in stage I (pT1) EOC was approximately 5-20% [1-6]. Reported rates of lymph node metastasis in CCC and serous cystadenocarcinoma (SAC) were summarized in Table 1[2-14]. From the results investigating a large number of CCC cases, retroperitoneal lymph node metastasis was observed in 9% in pTIa tumors, 7% in pTIc tumors, and 13% in pT2 tumors in CCC, which suggested that incidence of lymph node metastasis in CCC was lower than that of SAC [9]. Based on the subtotal of reported cases with pT1 and pT2 tumors, approximately one half incidence of lymph node metastasis in CCC in comparison with SAC was confirmed: 11% in CCC, and 25% in SAC.

**Table 1:** Rates of lymph node metastasis in early-staged clear cell carcinoma and serous adenocarcinoma

author	year	number of patients	pT stage	metastatic rate
clear cell carcinoma				
Di Re[2]	1989	11	pT1	9% (1/11)
Petru[3]	1994	2	pT1	0% (0/2)
Onda[4]	1996	16	pT1/2	31% (5/16)
Baiocchi[5]	1998	21	pT1	5% (1/21)
Suzuki[6]	2000	9	pT1	11% (1/9)
Sakuragi[7]	2000	23	pT1/2	17% (4/23)
Negishi[8]	2004	46	pT1	12% (5/42)
			pT2	75% (3/4)
Takano[9]	2006	173	pT1a	9% (3/36)
			pT1c	7% (7/99)
			pT2	13%(5/38)
Harter[10]	2007	7	pT1	0% (0/7)
Desteli[11]	2010	4	pT1	0% (0/4)
Nomura[12]	2010	36	pT1/2	6% (2/36)
**Subtotal**		**348**		**11%(37/348)**
Serous cystadenocarcinoma				
Di Re[2]	1989	40	pT1	28% (11/40)
Petru[3]	1994	21	pT1	38% (8/21)
Onda[4]	1996	21	pT1/2	33% (7/21)
Baiocchi[5]	1998	106	pT1	26% (27/106)
Suzuki[6]	2000	13	pT1	31% (4/13)
Sakuragi[7]	2000	25	pT1/2	8% (2/25)
Morice[13]	2003	26	pT1	31% (8/26)
Negishi[8]	2004	35	pT1	4% (1/24)
			pT2	36% (4/11)
Harter[10]	2007	13	pT1	15% (2/13)
Desteli[11]	2010	7	pT1	14% (1/7)
Nomura[12]	2010	12	pT1/2	50% (6/12)
**Subtotal**		**319**		**25%(81/319)**

Lymphadenectomy is so important to detect metastatic lymph nodes, as the patients with positive lymph nodes had poorer prognosis. However, the role of lymphadenectomy remains unclear based on the therapeutic aspect. Several authors reported that lymph node metastasis is independent prognostic factor for CCC [7,8,15]. Magazzino et al. analyzed 240 CCC retrospectively and reported as followed [15]: (1) Of 240 cases, 47.9% had lymphadenectomy and most of cases received platinum based chemotherapy after primary surgery. (2) The cases who received lymphadenectomy had longer progression-free survival (PFS) than the cases who had no lymphadenectomy in stage I/II, III/IV and all stage (p = 0.0258, p = 0.00337, p = 0.0001). (3) In advanced cases, lymphadenectomy prolonged the overall survival (OS). (4) In CCC, lymphadenectomy and clinical stage are independent prognostic factors by multivariate analysis. However, we reported that pN status showed only a marginal significance upon PFS and no significance upon OS based on the analysis of 199 CCC [16]. Other reports failed to show the usefulness of lymphadenectomy as prognostic factor [17,18]. Further examination will be required to confirm the role of lymphadenectomy for CCC.

In our studies, multivariate analysis revealed that peritoneal cytology status was independent prognostic factor for PFS (p = 0.04), but not for OS, and in addition, completion of surgical staging procedures was not a prognostic factor [16]. Higashi et al. analyzed 224 CCC patients with stage I and reported as followed [19]: (1) there was no significant difference in both OS and PFS of CCC between stage IA and IC (intraoperative capsule rupture), and the 5-year OS rate of stage IC(intraoperative capsule rupture) CCC patients was comparable to those with the non-CCC. (2) Stage IC CCC patients except for IC (intraoperative capsule rupture), such as positive ascites/washing and capsule surface involvement, had a poorer OS and PFS than those with IC (intraoperative capsule rupture). The results suggested stage I CCC cases other than intraoperative capsule rupture were at a considerable risk for recurrence and mortality.

Finally, the role of complete surgical staging still remains unclear for CCC. Several reports demonstrated that adjuvant chemotherapy had little impact on the survival of stage I CCC patients [16,20]. From these findings, complete surgical staging procedures are required at least to detect high-risk patients of recurrence; however, the extent of the surgery could not improve overall survival of CCC.

### Cytoreductive surgery

Optimally cytoreduced patients of EOC were reported to show a significant survival benefit over those patients who are suboptimally debulked, and there is a significant survival advantage in patients who are able to be debulked to less than 1 cm of residual disease. Hoskins et al. reported that patients with clear cell and mucinous histology had poor outcome even when they had small residual tumor after primary surgery [21]. We previously reported that there is no significant prognostic difference between the patients with the tumor diameter less than 1 cm and those with the tumor diameter more than 1 cm, and complete surgery is only the independent prognostic factor [9]. Kennedy et al. reported that among patients with advanced stage cancers (FIGO stages III and IV), CCC patients were more often optimally debulked than non-CCC patients (60% vs. 37%, p = 0.033) [22]. From these findings, the goal of primary surgical treatment for CCC may be complete resection.

### Fertility-sparing surgery

Fertility-sparing surgery (FSS) for reproductive-age patients with EOC has been adopted for stage IA and non-clear cell histology grade 1 (G1)/grade2 (G2) according to the 2007 guidelines of the American College of Obstetrics and Gynecology (ACOG) and unilateral stage I tumor without dense adhesions showing favorable histology (ie, non-clear cell histology G1/G2) according to the 2008 guidelines of the European Society for Medical Oncology (ESMO). In Japan, stage IA tumor or unilateral stage IC tumor on the basis of intraoperative capsule rupture and favorable histology are candidate for FSS according to the 2010 guidelines of the Japan Society of Gynecologic Oncology (JSGO). These 3 guidelines commonly eliminate CCC for the candidate of FSS. In contrast, in the 2010 guidelines of the National Comprehensive Cancer Network (NCCN), a stage I patient with CCC is an acceptable candidate for FSS. For the patients to receive FSS, randomized study cannot be performed because of ethical aspect. In this review, we summarize the FSS for CCC based on the retrospective studies.

Schilder et al. demonstrated that no recurrence was observed among 5 patients with stage IC CCC who received FFS; however, the detail of stage or postoperative chemotherapy was not recorded [23]. Kajiyama et al. reported the clinical outcome of 10 patients with stage I CCC treated with FSS (IA:4, IC(intraoperative capsule rupture): 5, IC(positive for malignant ascites):1) and demonstrated as follow [24]: (1) Among 10 patients, 9 patients received chemotherapy after surgery, (2) one patient with IC(positive for malignant ascites) who received postoperative chemotherapy recurred. Sato et al. reported 30 patients with stage I CCC who received FFS and reported as follow [25]: (1) Among 15 IA cases, 9 cases received chemotherapy after surgery and no one recurred, (2)Among 15 IC patients, 11 patients received chemotherapy after surgery, and 2 patients (IC(intraoperative capsule rupture):2) recurred among 11 patients who received chemotherapy and 3 patients (IC(intraoperative capsule rupture):2, IC(positive for malignant ascites or surface capsule involvement):1) recurred among 4 patients who did not received chemotherapy. (3) Recurrent sites are residual ovary (n = 3), lymph node (n = 2), peritoneum (n = 2) and liver (n = 1). (4) The 5-year survival rate is 93.3%. These data are shown in Table 2.

**Table 2:** Relapse rates of clear cell carcinoma patients who received FSS

stage	author	year	number of patients	relapse
Stage IA	Kajiyama [23]	2008	4	0% (0/4)
	Satoh [24]	2010	15	0% (0/15)
	total		19	0% (0/19)
Stage IC	Schilder [22]	2001	5	0% (0/5)
	Kajiyama [23]	2008	6	17% (1/6)
	Satoh [24]	2010	15	33% (5/15)
	total		26	23% (6/26)

We summarized Kajiyama’s and Sato’s reports in detail: (1) Among 19 patients, 12 patients received postoperative chemotherapy and no one recurred. (2) Among 21 IC patients, 17 patients received postoperative chemotherapy, and recurrent rate of IC(intraoperative capsule rupture) and IC(positive for malignant ascites or surface capsule involvement) are 25%(4/16) and 40%(2/5). (3) Among 17 IC patients who received postoperative chemotherapy, 3 (18%) patients recurred and among 4 IC patients who did not received chemotherapy, 3 (75%) patients recurred.

Recently, Kajiyama et al. also analyzed the OS of 16 patients with stage I CCC who underwent FSS and compared survival with 204 patients receiving radical surgery, or 64 patients with non-CCC undergoing FSS and demonstrated that patients with CCC who underwent FSS did not show a poorer survival than non-CCC patients who underwent FSS, or those at the corresponding stage with no CCC [26].

From these findings, CCC IA patient may be candidate for FFS and postoperative chemotherapy may be useful for CCC IC patient who received FFS.

## Chemotherapeutic treatment

Clear cell carcinoma (CCC) is a quite unique ovarian tumor showing resistance to platinum-based chemotherapy. The effect of the gold standard therapy for ovarian carcinomas, combination with paclitaxel and carboplatin (TC), is not satisfactory for CCC. Irinotecan hydrochloride, a topoisomerase I inhibitor, is a candidate for the treatment for CCC. Irinotecan combined with cisplatin (CPT-P) has been recognized to have an activity no less than TC for CCC. A world-wide prospective clinical study to compare CPT-P and TC as the first-line chemotherapy for CCC, GCIG/JCOG (Gynecologic Cancer Intergroup/Japanese Gynecologic Oncology Group) 3017, is now ongoing. Additionally, molecular-targeting agents are evaluated for advanced or recurrent CCC. We would discuss the chemotherapeutic regimens as primary or second-line therapy for CCC in this review.

### Primary chemotherapy using cytotoxic agents

It has been implied that CCC of the ovary showed resistance to conventional platinum-based chemotherapy [27-29]. Recent studies have confirmed the evidence in the analysis of patients with measurable CCC. Objective response was observed in 11-27% with conventional platinum-based regimen, whereas patients with serous adenocarcinoma (SAC) subtype showed a significantly higher response rate of 73-81% [30-32]. A report showed survival benefit of conventional chemotherapy with paclitaxel and platinum after complete surgery in CCC patients [33]. However, the result from large series of CCC patients treated with paclitaxel and platinum showed no survival benefit compared with conventional platinum-based chemotherapy in both early and advanced cases [9]. The results suggested that TC therapy, which is commonly used for ovarian carcinoma, is not effective enough for CCC patients. Reported response rates of primary therapy for CCC are summarized in Table 3[9,29-33].

**Table 3:** Response rates of primary chemotherapy for clear cell carcinoma

regimen	author	year	response/ Number of patients, response rate
Conventional Platinum-based	Goff [28]	1996	1/6, 17%
	Sugiyama [29]	2000	3/27, 11%
	Ho [30]	2004	4/15, 27%
	Takano [9]	2006	5/30, 17%
Taxane-Platinum	Enomoto [31]	2003	2/9, 22%
	Ho [30]	2004	9/16, 56%
	Utsunomiya [32]	2006	8/15, 53%
	Takano [9]	2006	9/28, 32%
Irinotecan-cisplatin	Takano [9]	2006	3/10, 30%

Irinotecan hydrochloride, a semisynthetic derivative of camptothecin, has additive and synergic effects in combination with cisplatin *in vitro*[34,35]. The combination therapy with irinotecan hydrochloride and cisplatin (CPT-P) was reported to be effective for patients with various solid tumors. Especially, a large clinical trial revealed that CPT-P had significant activity for extensive small-cell lung cancer [36]. Additionally, CPT-P had been reported to be effective in first-line and second-line chemotherapy for the treatment of CCC of ovary [37,38]. A large retrospective analysis indicated that CPT-P had a potential therapeutic effect at least no less than TC therapy [39]. A phase II study (JGOG3014) to compare CPT-P and TC for first-line treatment for CCC was conducted. The study revealed that completion rate of six cycles and five-year progression-free survival was similar in both arms [40]. Interesting to note, in the patients with residual tumor less than 2 cm, overall survival was marginally improved in CPT-P group in comparison with TC group (p = 0.056). Subsequently, a phase III randomized study to compare CPT-P and TC as adjuvant chemotherapy for CCC is on-going (GCIG/JGOG3017) [41]. The winner regimen will be the first regimen for histologically individualized therapy for ovarian cancers.

Another issue concerning chemotherapy for CCC is adjuvant therapy for patients with stage I disease. CCC is regarded as grade 3 tumor, and clinical guidelines recommend adjuvant chemotherapy for all patients with CCC, even at stage Ia. A large retrospective analysis of stage I CCC revealed that there were no statistical differences of progression-free survival (PFS) and overall survival (OS) between patients with chemotherapy and without chemotherapy [16]. Also, multivariate analysis showed that peritoneal cytology status (p = 0.02) and pT status (p = 0.04) were independent prognostic factors for PFS, however, adjuvant chemotherapy was not a prognostic factor (p = 0.80). The results suggested adjuvant chemotherapy had little impact upon survival of stage I CCC patients. Further strategy, such as a molecular targeting agent, is needed to improve survival of CCC, especially cases with positive peritoneal washing.

### Second-line chemotherapy for CCC

In a large series of platinum-sensitive relapsed ovarian tumors including all histological subtypes, overall response was 54% of the patients treated with the conventional platinum-based chemotherapy, and 66% of the cases treated with paclitaxel plus platinum chemotherapy [42]. In the platinum-resistant tumors, however, response rate using anti-cancer agents usually range from 25 to 30% [43]. In the second-line or salvage settings, the response rate for recurrent or refractory CCC was extremely lower than that for other histological tumors: even in the patients with platinum-sensitive CCC disease, the response rate reported was lower than 10% [44,45]. So, we have summarized reported cases that achieved objective response (Table 4) [30,33,44-48].

**Table 4:** Response rates of salvage chemotherapy for recurrent or refractory clear cell carcinoma

regimen	author	year	response/ number of patients, response rate
Megestrol acetate	Walailak [45]	2001	2/10, 20%
Cyclopshosphamide+cisplatin	Takano [46]	2008	1/9, 11%
Irinotecan+Platinum	Sugiyama [29]	1998	1/3, 33%
	Takano [46]	2008	2/15, 13%
Etoposide+Platinum	Takano [46]	2008	2/13, 15%
Paclitaxel+Carboplatin	Utsunomiya [32]	2006	3/13, 23%
	Crotzer [43]	2007	2/7, 29%
Gemcitbine	Crotzer [43]	2007	1/9, 11%
	Yoshino [47]	2012	1/5, 20%
Docetaxel+Irinotecan	Yoshino [47]	2012	1/11, 9%
Temsirolimus	Takano [46]	2011	1/5, 20%

Recently, single agent gemcitabine could be a candidate for salvage therapy for CCC, as the authors suggested [44,48]. Other regimens that showed objective response included irinotecan/platinum, etoposide/platinum, and paclitaxel/carboplatin; however, the efficacy was limited with progression-free interval approximately 6 months. Despite importance of response, it would be more important to monitor if adverse effects of chemotherapy worsen quality of life of the patients. Among these reports, the longest progression-period of 14 months was obtained by Temsirolimus [47]. The observed response duration was surprisingly longer than those obtained by any cytotoxic agents so far with no serious toxicities. The report encouraged us to investigate another chemotherapeutic strategy for CCC.

From the reported cases, however, it could be concluded that CCC is a potentially extremely chemo-resistant tumor against cytotoxic agents, especially in recurrent or refractory settings. Another strategy including molecular targeting agents might be needed for the treatment of these tumors.

### Incorporation of molecular targeting agents for the treatment of CCC

In the aspects of molecular characteristics as well as clinical behavior, it is hypothesized that CCC belongs to a different entity from other histological subtypes of ovarian carcinoma. First of all, the incidences of p53 mutation and p53 overexpression were much less frequent in CCC than in other histologic types of epithelial ovarian cancer [49,50]. On the other hand, mutation of p53 gene was quite frequent in serous subtype of ovarian cancers, and most of the alterations were missense mutations [51]. In addition to p53 status, CCC has a quite unique expression pattern of several molecules. Glutathione peroxidase 3 (GPX3) was found at levels 30-fold higher on average in CCC compared with the other ovarian cancer subtypes through studies with cDNA arrays and serial analysis of gene expression [52]. Elevated expression of GPX3 might contribute to chemoresistance phenotype, which is often observed in the patients with CCC. Another investigation using oligonucleotide microarrays reported that glutaredoxin (GLRX) and superoxide dismutase 2 (SOD2), in addition to GPX3, were highly expressed in clear cell type ovarian cancer, suggesting that high levels of these proteins relating with antioxidant function render CCC to be more resistant to chemotherapy [53,54].

Further, a report using oligonucleotide probe arrays showed that a transcription factor, hepatocyte nuclear factor-1 (HNF-1) was upregulated in CCC cell lines [55]. Overexpression of HNF-1 was confirmed by immunohistological staining of clinical samples. Further, overexpression of HNF-1 was observed in the specimens of borderline clear cell tumor and benign clear cell tumor [56]. The expression of HNF-1 was detected in not only atypical endometrial tissue, but also in endometriosis with degenerative and regenerative changes, suggesting that early differentiation into the clear cell lineage takes place in the endometriotic epithelium, and HNF-1 contributes to carcinogenesis of CCC.

Recently, immunohistochemical analysis showed that hypoxia-inducible factor 1 alpha (HIF-1alpha) expression levels were significantly higher in CCC than in other histological types of ovarian cancers [57]. Upstream target of HIF-1alpha, mammalian target of rapamycin (mTOR), was also reported to be up regulated in CCC [58,59], which was selected for molecular target of CCC.

There are two international collaborating studies led by Gynecologic Oncology Group (GOG) to evaluate efficacy of molecular targeting agents for CCC of the ovary [60,61]. It is true that there existed super-responders against molecular targeting agents in the patients with CCC. Consequently, further studies to evaluate these new drugs should include biomarker analysis to predict response or adverse effect for clinical application.

## Conclusions

CCC has unique characteristics among ovarian cancers. We have to deal with the tumor using completely different techniques of treatment modality in terms with surgery and chemotherapy. Especially, we have to focus on histology-specific features of molecular pattern. We hope the day will come when CCC tumors would be easily handled by the selection of effective surgery and chemotherapy including molecular targeting agents.

## Abbreviations

CCC: Clear cell adenocarcinoma; SAC: Serous cyst adenocarcinoma; EOC: Epithelial ovarian carcinomas; PFS: Progression free survival; OS: Overall survival; FSS: Fertility-sparing surgery; ACOG: American college of obstetrics and gynecology; ESMO: European society for medical oncology; JSGO: Japan society of gynecologic oncology; NCCN: National comprehensive cancer network; GCIG: Gynecologic cancer intergroup; JCOG: Japanese gynecologic oncology group; CPT-P: Irinotecan hydrochloride + cisplatin; TC: Paclitaxel + carboplatin; GPX3: Glutathione peroxidase 3; GLRX: Glutaredoxin; SOD2: Superoxide dismutase 2; HNF-1: Hepatocyte nuclear factor-1; HIF-1: Hypoxia-inducible factor 1; mTOR: Mammalian target of rapamycin; GOG: Gynecologic oncology group.

## Competing interests

The authors declare that they have no competing interests.

## Authors’ contributions

Dr Takano and Dr Tsuda wrote the manuscript. Dr Takano, Dr Tsuda, and Dr Sugiyama approved it. All authors read and approved the final manuscript.
